# Aerosolize this: Generation, collection, and analysis of aerosolized virus in laboratory settings

**DOI:** 10.1371/journal.ppat.1011178

**Published:** 2023-03-09

**Authors:** Jessica A. Belser, Joanna A. Pulit-Penaloza, Taronna R. Maines

**Affiliations:** Influenza Division, Centers for Disease Control and Prevention, Atlanta, Georgia, United States of America; University of Wisconsin-Madison, UNITED STATES

Airborne transmission of viral pathogens is dependent on the generation, exhalation, and deposition of virus-laden aerosols from infected to susceptible hosts. Rigorous evaluations of virus transmissibility in laboratory settings have provided critical insight into viral and host features that contribute to this property, but historically have not included concurrent evaluations of viral load in the air. Similarly, investigation of respiratory pathogens in an aerosol state has been understudied relative to other areas focused on transmission dynamics between mammalian hosts. What makes collection and quantification of virus-laden aerosols in the laboratory so challenging? Here, we discuss practical obstacles and limitations on performing this work in laboratory environments, the additional challenges posed by conducting these experiments concurrent with in vivo experimentation, and how continued investment in this work will provide greater understanding of the role aerosols play in viral transmission.

## Transmission at any size

Humans release respiratory droplets following various activities, from breathing and speaking, to coughing and sneezing, and even singing and playing wind instruments. These droplets vary in size (from submicron diameters to droplets visible to the human eye) and are emitted at variable levels and momentum depending on the respiratory action and individual person [1]. In general, respiratory droplets contain a mix of water, inorganic substances, and proteins [2] as well as pathogens if emitted from an infected host; these droplets are subject to evaporation, leading to shrinkage and longer persistence in the air compared to droplets of the original/initial size. Importantly, infection can affect the number, size, and composition of expelled droplets relative to healthy individuals, which collectively modulate the distance traveled, viability of the virus within the droplets, and deposition location if inhaled by a susceptible host [3]. This complexity represents a substantial challenge for rigorous study, and as such, efforts in laboratory settings to measure the role virus-laden aerosols play in transmission events typically focus on one of these properties at a time.

Numerous small mammalian models are employed in laboratory settings to evaluate virus transmissibility by the airborne route [3]; these models separate donor and contact animals to prevent direct or indirect contact, while only permitting air exchange between cages, with or without directional airflow. These stringent models thus implicate virus-laden aerosols as the only source of infectious virus to which the contact animals are exposed, but infrequently include collection and quantification of viral particles released by infected animals. However, inclusion of these assessments has provided critical insight into the role particle size contributes to onward transmission [4–6]. Quantification of total particles emitted into the air is often achieved via use of an aerosol particle sizer, which permits total aerosol counts at different size bins, but does not preserve collected aerosols for subsequent analysis [4,7].

## Not all laboratory-generated aerosols are created equal

**Fig 1:**
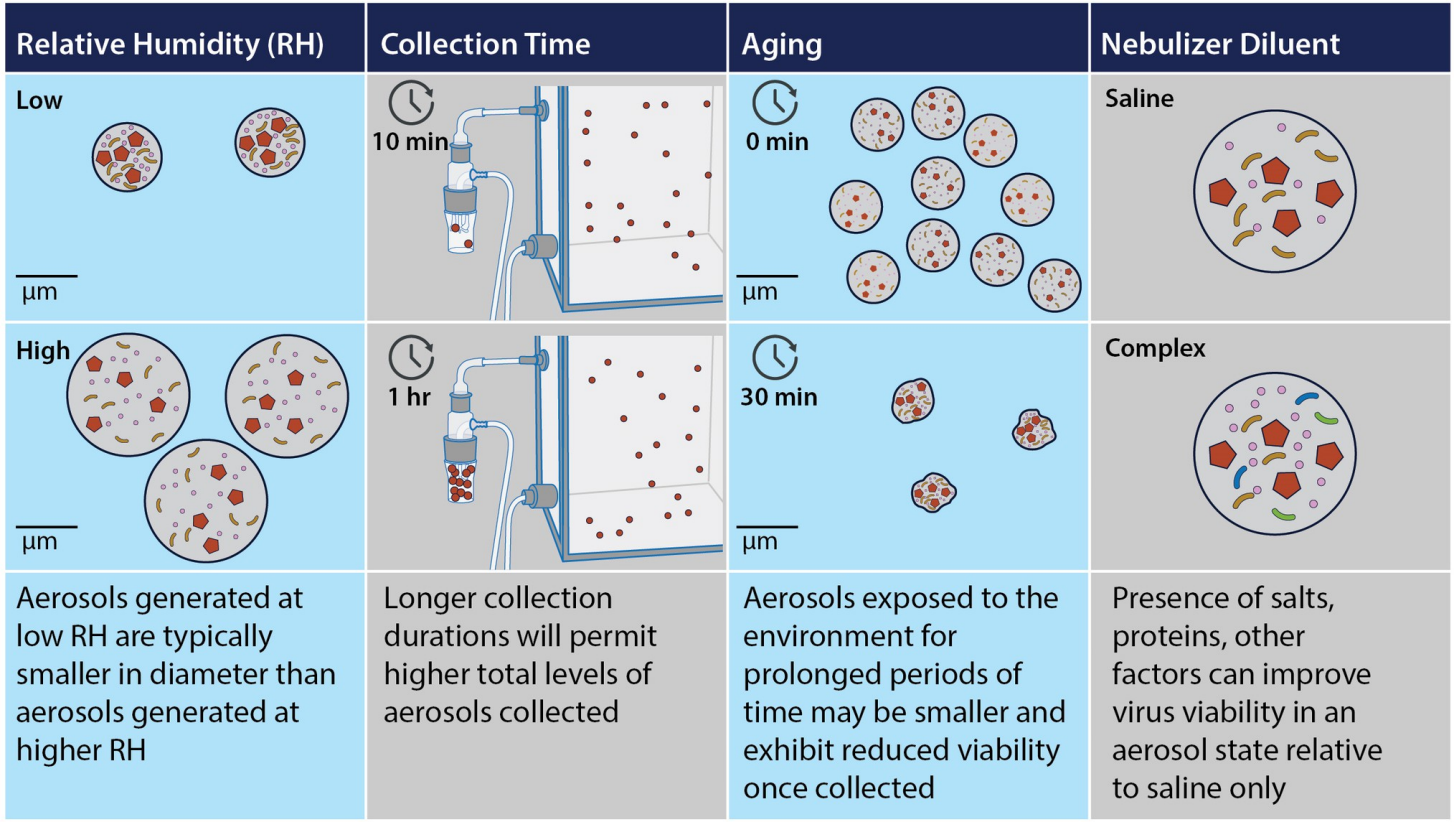
Examples of protocol-specific parameters that can modulate properties of aerosols generated in the laboratory. Columns depict some of the specific variables that may be controlled for and manipulated in laboratory settings. Within particles, red pentagons depict virus being aerosolized, circles and rods depict salts and proteins, respectively, with rods of different colors depicting a greater diversity of proteins within more physiologically relevant diluent suspensions. Scale bars and time intervals are for representative purposes only. The parameters presented here can influence each other (e.g., aging aerosols at different humidities may differentially alter particle size), and summary statements represent one of several potential outcomes from modulating these (and other) experimental conditions.

Aerosols can be generated from liquid suspensions of virus in controlled laboratory settings but can vary widely based on equipment and established procedures between laboratories. The choice of nebulizer and sampler influences the properties of aerosols generated and recovered, respectively, as well as preservation of virus viability [8,9]. The most rigorous aerosol generation and collection systems need to balance real-time monitoring of parameters, control of airflow, and environmental conditions throughout experimentation, in tandem with safety controls to ensure all infectious material is subsequently inactivated so that laboratorian safety is prioritized [10]. A multitude of protocol-specific parameters can modulate properties of aerosols generated and collected in the laboratory, including temperature and humidity, diluent composition in the nebulizer and sampling device, collection time, the duration and/or manner in which aerosols are aged, and airflow through the system (Fig 1), complicating interpretation of results between laboratories. Side-by-side comparisons of these variables can be extremely valuable but are not often conducted [11,12].

## Collected at any cost

In laboratory settings, aerosolization of liquids containing high concentrations of infectious virus permits the recovery of high levels of infectious virus in a sampler. In the context of in vivo experimentation, infectious virus is emitted from infected animals at much lower titers, necessitating collection methods that permit sampling for prolonged periods of time, employing low volume suspensions for collection to concentrate virus, maintaining cooler temperatures in the sampler, and employing other design features to preserve virus viability. Therefore, measurement of airborne virus shedding from infected animals during transmission assessments most often involves reporting viral nucleic acid and not infectious virus [4–7].

There are numerous practical, logistical, and methodological reasons that contribute to reporting of viral genome copies and not infectious virus [13]. Levels of genomic material are typically several orders of magnitude higher than infectious virus levels [5,7]. Samplers are generally inexpensive, easy to use and decontaminate, and may be operated for long periods of time without the need for hands-on laboratory staff or direct manipulation of infected animals [4–6]; however, they often are not designed to preserve virus structure and infectivity. As such, mechanical shearing and desiccation of virus precludes accurate measurement of infectious virus in the air, allowing only for quantification of viral genomic material [8,9]. Furthermore, since small animal models emit relatively low minute volumes of air into often large animal housing areas, extended (>1 hour) sampling windows may be necessary to collect detectable levels of virus-laden aerosols diluted in large volumes of air, especially if aerosols are being size-fractionated into multiple populations; longer collection durations can compromise virus viability prior to optimal storage and infectious virus quantification. Lastly, specimen handling post-collection routinely involves a freeze–thaw step, decreasing recovery of infectious virus. For these reasons, efforts to incorporate aerosol collection into routine, labor-intensive, and time-consuming laboratory procedures and pandemic risk assessment activities have been most successful when using simple sampling techniques allowing for storage of samples for analysis outside of the workflow, resulting in viral genome detections as a primary readout. These studies nonetheless provide valuable contextual information regarding the kinetics and magnitude of virus emission into the air, linking these features with the frequency and timing of virus transmission between infected animals and susceptible contacts [4–6].

## It’s alive!

Collection of infectious viruses in aerosols is most desirable, but as discussed above, there are a multitude of practical issues that preclude their routine quantification, especially during in vivo settings. For sampling devices that require animal restraint and/or sedation for optimal collection (e.g., direct collection of animals’ breath into the sampler to avoid aerosol diffusion into the environment during collection), sampling durations are limited by anesthesia schedules. Staging sensitive collection devices within reach of an awake and alert animal can be impractical or impossible if the device is not designed for such wear-and-tear (e.g., contain parts that can be consumed by animals); not all caging or animal restraint systems can support close staging of samplers that require a dedicated resting surface while concurrently prohibiting direct interaction between the device and curious animals. Transmission experiments are frequently conducted with 3 to 4 pairs of donor:contact animals housed in different cages [5,6,14]; should concurrent sampling be desired from all animals within the same time frame, this requires multiple instruments and associated equipment, which can be cost- or space-prohibitive.

Although multiple samplers have been shown to efficiently preserve infectivity following collection of laboratory-generated aerosols containing high levels of virus [15], collection of infectious viruses during in vivo experimentation is less efficient due to the low levels of virus emitted into the air, and the potential for virus viability to decrease following prolonged sampling. For example, infectious virus was collected from influenza virus–infected ferrets following tidal breathing or induced sneezing via a cascade impactor [7], but at much lower titers than following aerosol generation using a Collison nebulizer [12]. Samplers employing water-based condensation have been gaining interest as they employ gentle collection methods resulting in concentrated infectious virus more effectively relative to other methods [16,17]. However, there is still a need for development of sampling devices, at a range of price points, which balance the benefits of preserving virus viability alongside the scalability and flexibility for routine use in laboratory settings, especially during in vivo experimentation (Table 1).

**Table 1:** Laboratory approaches to sample aerosols released from virus-infected animals.

**Aerosol sampler device** ^**a**^	**Advantages**	**Disadvantages**
**Cyclone sampler (e.g., 2-stage NIOSH)**	Permits size fractionation of particles, samples unobtrusively collected from unanesthetized animals, easily scalable for multiple concurrent sample collections, wide range of sample duration times possible, no animal handling needed, high collection efficiency	Not designed for recovery of infectious virus
**Cascade impactor (e.g., Andersen impactor)**	Suitable for recovery of infectious virus, permits size fractionation of particles, high laboratorian control over number of fractions sampled, and type of collection material employed	Challenging to employ with unsedated animals, collection time limited by duration of anesthesia of animals being sampled, can be labor-intensive for laboratory staff, reduced efficiency of capturing viable virus when at low concentrations
**Liquid impinger (e.g., SKC Biosampler)**	Suitable for recovery of infectious virus, includes multistage liquid impingers (MSLI), permits size fractionation of aerosol particles	Challenging to employ with unsedated animals, high volume of liquid can dilute low concentration samples to undetectable levels, can be labor-intensive for laboratory staff, evaporation of liquid during prolonged collection durations is possible, low collection efficiency for particles <0.3 μm
**Condensation sampler (e.g., viable virus aerosol sample, VIVAS)**	Designed for recovery of infectious virus, low sample volume	Only one animal sampled at a time with a single device, does not permit size fractionation of aerosol particles, large instrument size
**Filter samplers (e.g., polytetrafluoroethylene, PTFE)**	Recovery of infectious virus is possible depending on filter material, aerosols exposed to large surface area, samples unobtrusively collected from unanesthetized animals, easily scalable for multiple concurrent sample collections	Not designed for recovery of infectious virus, filter material can lead to virus desiccation, does not permit size fractionation of aerosol particles
**Aerosol particle counter (e.g., TSI Aerodynamic Particle Sizer, TSI Aerotrak)**	Permits quantification of total number and size of aerosol particles released from an animal	Not designed for recovery of infectious virus, does not distinguish virus-laden aerosol particles from all particles collected, only one animal sampled at a time with a single device

^a^Content sourced from [4–9,11,13,15–17].

## Moving forward

Despite the challenges highlighted above, there is a need to better define the role of aerosols in laboratory-based viral transmission assessments to discover pearls of wisdom that can translate into substantive benefits to public health. To this end, recent comparative studies have assessed the relative impact strain-specific, diluent-specific, environmental condition–specific, and device-specific changes confer to virus aerosolization [18,19]. Laboratory-generated aerosols cannot fully emulate the true complexity of airborne particles exhaled from mammalian hosts, but efforts to better understand virus behavior within these particles under defined conditions nonetheless improve our ability to extrapolate results to real-world settings, such as sampling in agricultural environments [20].

Wider adoption of aerosol collection during in vivo assessments of virus transmissibility would be of benefit. Beyond current efforts of quantifying airborne virus released from infected animals and linking these data to virus transmissibility [4–6], future efforts to obtain genomic sequence data from airborne virus will facilitate bridging of within-host and between-host evolution and transmission dynamics. Furthermore, most virus transmission assessments reported have been conducted in serologically naïve, healthy animals; expansion of studies to include hosts with diverse immunological and/or health profiles to better elucidate how altered host states modulate release of virus-laden aerosols post-infection is needed. As laboratory-based transmission studies continue to play crucial roles in virus risk assessment efforts [8], continued inclusion of the role aerosols play in this dynamic process represents a necessary endeavor.
